# Population movements, borders, and Chagas disease

**DOI:** 10.1590/0074-02760210151

**Published:** 2022-07-08

**Authors:** Andrea Avaria, Laia Ventura-Garcia, Mariana Sanmartino, Carlos Van der Laat

**Affiliations:** 1 Universidad Autónoma de Chile, Facultad de Ciencias Sociales y Humanidades, San Miguel, Chile; 2 Universitat Rovira i Virgili, Medical Anthropology Research Center, Tarragona, Catalonia; 3 Consejo Nacional de Investigaciones Científicas y Técnicas, Instituto de Física de Líquidos y Sistemas Biológicos, Grupo de Didáctica de las Ciencias, La Plata, Buenos Aires, Argentina; 4 International Organization for Migration, Migrant’s Health Assistance Program Coordinator, Geneva, Switzerland

**Keywords:** migration, human mobility, Chagas disease, social determination

## Abstract

Currently, Chagas disease is a complex global health problem with local and global implications. In the present article, we approach this complexity from the perspective of human mobility and its effects on people’s health in places of origin and in transit and destination. We raise key concepts such as human mobility - understood as a possible socio-structural and economic determination of health -, the associated social and institutional barriers and the processes of social exclusion related to Chagas disease. We also propose what we identify as emerging opportunities from the perspective of health as a right. Finally, we propose strategies aimed at addressing Chagas disease from a multidimensional and intersectional perspective in complex, diverse and interconnected territories through migration.

Currently, Chagas disease is a health problem that affects at least 7 million people worldwide.^1^ However, its globalisation implies a paradox related to human mobility. As Farmer points out, “political borders serve as semi-permeable membranes, often quite open to diseases and yet closed to the free movement of cures”.^2^ Such paradox questions any attempt to approach the problem from a reductionist or universal perspective and urges the need to incorporate multidimensional views that recognise the numerous complexities, articulations and intersections that occur at local, regional and global levels.^3^ For these reasons, in this article we will refer to ‘Chagas’ as an alternative to the classical denomination of ‘Chagas disease’, with a broader meaning to include also the psychological, socioeconomic, anthropological, and other aspects that affect people infected by *Trypanosoma cruzi* (most of whom will never develop the disease), their relatives and societies.

The last 30 years have been characterised by a modification and increasing complexity in the epidemiological conditions and characteristics of Chagas. Environmental changes and increasing human mobility have been the turning points. Therefore, incorporating the recognition of this change in the understanding of the current epidemiological panorama of Chagas implies considering and jointly addressing biomedical, environmental, psychosocial, sociocultural, and political-economic components. To this end, it is essential to contribute perspectives that include the understanding of Chagas as a health-disease-prevention-care process^4^ from critical approaches, articulated with the affected people’s experiences, and contemplating the particularities of the territories and the constitution of the inequities that emerge around Chagas in different contexts.^5^ ^-^ ^11^ According to Sanmartino et al.,^12^ “it is crucial to support the fact that, beyond the biomedical and epidemiological aspects traditionally (and almost exclusively) considered, in the current and past configurations of Chagas disease converge an intricate web of elements related to the social, cultural, economic, political and environmental aspects, among others. Today, more than 100 years after its ‘discovery’, it is evident that the classical scenario in which it was described has changed and that, in a globalised context, we must overcome the biomedical dimension to improve people’s health conditions”.

The intersection between mobility and Chagas presents us with an opportunity to outline proposals aimed at guaranteeing living conditions that favor populations’ health and their access to health services in a globalised world. In this scenario, we ask ourselves: What are the challenges we face in relation to Chagas and human mobility? And, from a perspective of rights: What are the possibilities that Chagas poses in the generation of new ways of addressing complex health problems?

Based on this intersection, in this article we approach the problem of Chagas based on the experiences provided by migrants and affected groups - where social exclusions of class, gender, ethnicity, age or national origin, among others, converge - and we analyse the limitations and strengths of health systems, social policies and models for addressing the issue in a context of human mobility.

**Chagas and human mobility**

Chagas has historically been linked to vulnerable and excluded people in Latin America, especially those of rural origin where triatomines (insect vectors of *T. cruzi*) proliferate. However, population movements at local rural-urban, regional and - more recently - global levels as a result of migrations from the Latin American region to the USA, later to Europe, and then within the American continent have expanded the geographical distribution of the infection to territories where it was not previously known, turning Chagas into an emerging disease of growing biosanitary interest (Figure).^13^ ^,^ ^14^ ^,^ ^15^ ^,^ ^16^ ^,^ ^17^ ^,^ ^18^ Social and/or economic crises in Latin America, armed conflicts or the consequences of the application of neoliberal policies in the region, among others, have contributed to delimit this new epidemiological scenario. In addition, it is estimated that currently about two thirds of people with Chagas live in urban areas inside and outside Latin America.^19^ This new scenario also poses a health, economic, social, and political challenge linked to human mobility, both internal and across borders. The settlement of populations in the urban periphery - where housing and sanitation conditions are generally precarious - and the lack or insufficiency of health services increase inequalities.^20^ In this context, identifying the social determinants of health allows highlighting the health-disease-prevention and care processes that underlie the causes of health inequalities between different social and cultural groups, based on social class, gender, age, ethnicity or territory.^4^ ^,^ ^21^ Consequently, it is pertinent to ask how the social and historical contexts characteristic of Chagas reproduce these inequalities and how they condition health status and access to health services among those groups that are in a process of local, regional, or international mobility.

**Figure:**
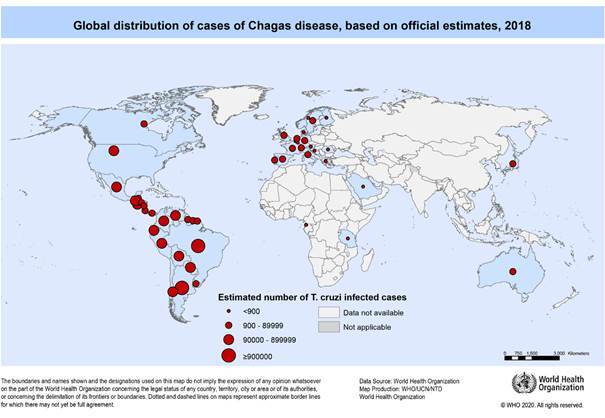
Global distribution of cases of Chagas disease based on official estimates, 2018. Source: OMS (2020). Available from: https://www.who.int/docs/default-source/ntds/chagas-disease/chagas-2018-cases.pdf?sfvrsn=f4e94b3b_2.

According to the International Organization for Migration (IOM),^22^ the relationship between health and migration implies recognising the conditions of vulnerability faced by migrants, the “inadequate access to health services and unfavorable conditions many migrants live and work in make them subject to a variety of health risks. [...] Mitigating health risks of migration and ensuring equitable access to health services for migrants and their families are important aspects of migration management. These measures are needed to improve the health status and overall well-being of migrants, reduce migrant vulnerability, protect global public health, facilitate integration and contribute to social and economic development”.^22^ Furthermore, we should bear in mind that the management of the health and disease of migrants and their families usually takes place, in part, in the transnational context, and this is also the case for Chagas.^9^ ^,^ ^10^ The transnational context is defined by Glick-Shiller et al.^23^ as “the emergence of a social process in which migrants establish social fields that cross geographic, cultural and political borders. Immigrants are understood to be transmigrants when they develop and maintain multiple relations - familial, economic, social, organisational, religious and political - that span borders”.^23^ This implies the need to include this space in the analysis, highlighting the importance of articulating the migratory cycle stages and the interconnections between contexts.

Human mobility is not a problem in itself, but rather of the processes that accompany it, which are generated and manifested, for example, in a series of conditions of inequality in health that require the incorporation of various actors, the development of policies and the generation of social and legal conditions for social inclusion.^24^ ^,^ ^25^ In turn, the processes of human mobility invite us to question the epistemological frameworks from which we usually understand reality, social subjects, health, disease and the search for health care.^12^ These new conceptual and epistemical frameworks challenge us to revise previous categories and analyses. This opens up the possibility of deconstructing commonly used concepts such as *tropical diseases*, *health transitions*, *national health profiles*, *endemic-non-endemic* countries/regions, *rural* disease, *poverty-associated* disease, to mention a few. Following Farmer,^2^ we understand that “models and even assumptions about infectious disease need to be dynamic, systematic and critical”.

**Chagas and borders: barriers and opportunities**

The resulting configuration between migration and Chagas is conditioned both by the axes of structural inequality - gender, social class, age, ethnicity, or territory, among others - as well as by the socioeconomic, cultural, and political contexts of countries and, in particular, by the laws that condition people’s mobility and access to rights such as work, housing, education or health, to give a few examples. Chagas is, after all, an expression of social, political, economic, and cultural conditions at local, regional and global levels, conditions that are often exacerbated during the stages and process of migration.

Human mobility evidences material, geographic and administrative borders as well as symbolic, social and subjective gaps. In this section, we will address the limitations faced by people who have migrated and the impact they have on the care and management of Chagas, both institutionally and for the affected groups. We will inquire into the implications that these borders, associated with human mobility, have with respect to Chagas. Finally, we will reflect on the opportunities that emerge for the health system, the receiving communities, and the people in mobility.

The narratives of people affected by Chagas represent a point of intersection with the stories of local, regional, and international mobility. For this reason, we have relied on the narratives of people of diverse nationalities to facilitate the understanding of the reflections that we develop. These accounts correspond to testimonies collected in various activities and previous research in Catalonia, Italy, and Chile. To preserve people’s identities, we have used fictitious names; to avoid stigmatisation linked to some of the countries of origin, we have also decided to omit this information in the shared testimonies.

*Mobility and inequalities. The territory as frontier, the “frontiers” of the territory* - As we mentioned, Chagas allows graphing the inequalities that occur in relation to inhabiting or moving between territories; it evidences the material and symbolic “frontiers” that emerge in the framework of Chagas issue and human mobility.

The difficulties arising from the conditions of migratory regularity or irregularity as well as the limitations to access health services and social and labor inclusion under conditions of rights and social protection (not always possible) have a clear impact on the general health of the migrant population.^24^ ^,^ ^25^ ^,^ ^26^ Likewise, changes in the forms of work, climate change, violence, increased poverty, political and social crises, and their effect on migration (internal, international, circular), the feminisation of migration, increased controls and restrictions on human mobility, to name a few, have led, among other consequences, to a change in migratory flows (in recent decades from south-north migration to south-south migration). In the current pandemic context, these changes have increased tensions in the territories and have further affected the rights of people in mobility.

Liberona^27^ states that, by restricting mobility, migratory policies in the Latin American context, instead of reducing it, have contributed to irregularity and have aggravated it. Border closures only exacerbate violations of people’s rights and increase racialisation, xenophobia, stigmatisation, and precariousness of those who migrate. The current pandemic, as never before, highlights the particular vulnerability faced by migrant and refugee.^28^ These conditions have also relegated pathologies such as Chagas to the background, thus further deepening its historical invisibilisation.

In particular, people affected by Chagas share with people in mobility conditions of inequality and structural inequity: stigmatisation, social exclusion associated with the condition of migrant, woman, peasant or indigenous, as well as differences in access to rights (labor, housing, health, etc.). Therefore, limitations in the quality and opportunity of health services and the lack of policies and programs for the detection, treatment, care and follow-up of Chagas can occur in both countries of origin and countries of destination.

The existing gaps in the level of access to health rights between countries of origin and countries of destination can be seen as barriers that people observe in their own families. In some cases, having treatment in countries of destination opens a health gap for members of the same family or community, in contrast to those who stay in the country of origin without access to treatment. Maria’s case is eloquent; she receives treatment in Barcelona and sends money to her country of origin to ensure her family’s diagnosis for Chagas: “I did not come here to be sick. I came here [Barcelona] to work, I am taking care of a lady. I left my children in my mother’s care. I did not know I had Chagas. When I found out I thought of my children and my mother. I had such an inner sadness that turned into anxiety when I thought of my children there, not knowing if they would also have Chagas. Maybe I passed it on to them? Until I finally sent them money so that they could all get tested, and I am thankful that none of them turned out to be sick” (María, migrant woman in Catalonia).

The inclusion of migrants in countries that guarantee health and care for Chagas opens the opportunity to find mechanisms to ensure the detection and treatment of the family left behind in the countries of origin. This double concern for the family’s health, at origin and destination, has strengthened collaboration between countries, building bridges between dissimilar realities that could be reinforced at the international level.^7^ ^,^ ^29^

On the other hand, migratory trajectories have an impact on the conditions and also on the perception of health and Chagas. This is especially complex when people have to deal with health systems with such differentiated guarantees of rights.^7^ ^,^ ^28^ ^,^ ^30^ In this framework, migratory circularity at regional level for reasons of temporary or seasonal work, especially between countries where Chagas has been considered “endemic”, requires particular attention since mobility between territories in irregular conditions prevents or interrupts the continuity of treatment or access to diagnosis and care.^31^

*The health system and people with Chagas: frontiers that emerge and install inequities* - The lack of knowledge about Chagas in the destination contexts emerges in migrants’ narratives. In health care scenarios, such as health or pregnancy check-ups, there is evidence of both explicit and implicit responses of unawareness on the part of health care teams. “My little doctor here [Barcelona] did not know what the disease [Chagas] was; when I told her that I had Chagas disease, she asked me: the disease of what?” (Gisselle, migrant woman in Catalonia).

The aforementioned lack of knowledge implies difficulties in the active search for Chagas among Latin American migrants.^9^ ^,^ ^10^ ^,^ ^32^ Limited access to serological tests, generally available at secondary (and not primary) levels, is also a difficulty. Not all countries, and even not all territories or services within the same country, ensure diagnosis and treatment for Chagas disease. The primary health care (PHC) level is key, among other reasons, to guarantee health check-ups for pregnant women, care for children and preventive health. PHC is closer to the population’s needs and is useful for the development of prevention and health promotion strategies according to local particularities, in addition to being a key link in the resolution of social inequalities. On the other hand, the rather inflexible schedules of health services, often incompatible with the long working hours and precarious working conditions of the migrant population, limit the access and use that migrant workers (both women and men) make of health systems. It is thus observed that, in general, the availability of services and actions are exclusively oriented to the needs and working conditions of health teams.^7^ ^,^ ^9^ ^,^ ^33^

In many countries, Chagas is not considered part of the national public health policy. This situation raises the need to expand screening in blood and organ donation and to pay special attention to the notification of positive cases, since the high mobility and precariousness faced by migrants limit the continuity of care processes. It is also essential to expand the detection of *T. cruzi* in women of reproductive age and especially in pregnant women and newborns and to ensure the treatment and follow-up of people affected by Chagas of any age.

On the other hand, specific mobility situations such as the adoption of Latin American children constitute a challenge for both the host families and the treating medical teams, due to the lack of background or the lack of knowledge about Chagas.^7^ Likewise, the naturalisation of Chagas, as evidenced by David in his story, constitutes a health barrier. “Vinchucas accompanied us throughout our childhood; most of us were born and grew up in houses with them. We have known them for so long that we have forgotten them” (David, migrant man in Catalonia).

This approach, which he shares with other compatriots in the context of origin, could become a difficulty when facing the anamnesis and resolution of the health and disease conditions at destination. Consequently, health teams should be prepared and active in searching for information and detecting Chagas, considering the prevalence and eventual naturalisation of this disease by the Latin American migrant population, and should also be open to detecting cases, due to the possible transmission of *T. cruzi* by connatal and transfusion routes.

In contexts close to borders between countries, health issues, migration and Chagas converge as elements that can increase the exclusion condition of the population in mobility, particularly people of indigenous origin. Isolation, lack of services and the presence, in general, of health professionals trained under a biomedical perspective make it difficult for people to access their health rights. Community participation in these territories and coordination with the closest PHC levels - regardless of the national territory in which they are located - should be guaranteed, promoting at the same time the recognition of their own health agents and models. Specifically, considering the community participation of local leaders is fundamental in the approach and resolution of health problems of indigenous groups living across borders.^34^

*Being a migrant, being a woman, having Chagas: the “borders” (that are perceived) in the body* - Both being a Latin American migrant and being infected by the parasite that causes Chagas disease are factors of discrimination and social exclusion. In particular, stigmatisation associated with poverty manifests itself in experiences of racism and xenophobia. In this way, stigmatisation increases the fear of a diagnosis and of seeking support from health services. “When I was 14 years old the word Chagas rebelled and shamed me, I hid it and did not want to acknowledge it until I got pregnant; then, fear and guilt were added. But I lost my fear and freed myself; now I can say, without fear or shame, that I have Chagas. That gives me strength for people to know, to be encouraged and to be able to say: I have Chagas” (Hilda, migrant woman in Catalonia).

Gender turns out to be a determining factor and, in the case of Chagas, women experience the responsibility for the transmission of *T. cruzi* to their sons and daughters; this is experienced as guilt that becomes flesh in the body (carrier-sick) of the newborn.^7^ ^,^ ^35^ “If I had known that the treatment at birth would cure my child, the fear of my pregnancy would have been another fear, a fear without guilt. Knowing that the treatment cures your child, if your child is born with Chagas disease, reassures you” (Juana, migrant woman in Catalonia).

Affected people share the fear of a disabling disease and/or a silent or unexpected death that is often linked to their biographical experiences as well as to their productive role in the context of migration,^10^ also in the case of migrant women, mainly engaged in household work.^25^ ^,^ ^36^ Among women, there is also the fear of direct transmission of Chagas disease to their children and the fear of not being able to maintain their domestic units in the society of origin and/or destination, bearing in mind that it is also common for them to assume the financial support of their family groups on their own, since men are no longer involved in paternal commitments:^25^ ^,^ ^36^ ^,^ ^37^ “I am afraid even about the fact of starting a family, of saying, damn, what if I have children now and then I have to pass away when I am 40 years old? I do not want that my children would be the same way I was left without my father” (Laura, migrant woman in Catalonia).

Thus, the axes of concern differ in some aspects depending on gender systems; however, the fear of illness is a transversal axis. “I ask a lot because my father had Chagas in his heart and one of my brothers also has it, and I am afraid that my heart will also have it” (Iván, migrant man in Catalonia). “So, also, this disease does not tell you what the symptoms are... only a preventive test tells you whether or not [you have it], so ideally you should know ‘I have this disease and if I do not take care of it, it can attack this part of my body, even if it is very silent.’ So that is where my mother tells me that in my country this disease is not... it is not on a priority scale of hereditary diseases; it is very low; because it is not something that they tell you ‘you have Chagas, you are going to die tomorrow’ or ‘you have Chagas and you are going to live the rest of your life with this disease’, because as it is silent, until it attacks a part of your organism, you are not going to know” (Clara, migrant woman in Chile).

On the other hand, the possible transmission of the infection through blood donation is often experienced as a responsibility that falls on the donor: “I gave blood to one of my brothers and eight months later I found out that I had Chagas and that I would have passed it on to him anyway” (Luis, migrant man in Catalonia).

Fears are not only related to the direct consequences of the disease on people’s bodies but, as evidenced by the following treating physician’s account, to the fear of stigmatisation and labor exclusion.^10^ The experience among migrants in Italy, for example, underlines the need to jointly resolve the adequacy of the information given to people with Chagas to avoid stigma and exclusion.^37^ “They fear that the result of the examination on hospital/health center letterhead may be seen by their employer and put their job at risk: they prefer to know the result by phone and not to have it on paper. Many people do not tell the family doctor about the result for fear that he/she will talk about the problem and their employer will find out” (Lucila, physician in Italy).

Along these lines, the representations that link Chagas with poverty, which often prevail in interventions and among health teams, have a paradoxical impact, disassociating Chagas from those groups that are in a better socioeconomic position or from those belonging to certain nationalities, considering them free of being infected with *T. cruzi*. In addition, the same popular representation links Chagas to certain migrants, which makes it difficult to care for those who are at equal risk: “I did not know that I could get it because I did not consider myself so marginalised. Yes, we come from a humble family, but there are more humble people, there are more! (...). There are people who have lived in worse conditions and who supposedly are not (sick)!” (Lidia, migrant woman in Catalonia).

These representations increase the invisibilisation of Chagas and of the people who experience it and, due to the prejudices that derive from them, can hinder access to health care for some and for others.^9^ ^,^ ^10^ The experience in countries that have detected Chagas as an emerging problem has focused efforts on guaranteeing care for affected individuals and communities based on individual, family and community strategies, with a focus on prevention, detection, care and follow-up.^32^ ^,^ ^38^ ^,^ ^39^ ^,^ ^40^ ^,^ ^41^ These actions are mainly oriented to the management of connatal Chagas,^42^ ^,^ ^43^ and the development of strategies that consider the people affected through the empowerment of specific leaders or representatives and support the constitution of social organisations of people affected by Chagas.^44^ ^,^ ^45^ ^,^ ^46^ ^,^ ^47^ In these contexts, innovation has been achieved through the development of interventions focused on information, education and communication (IEC), in actions that are mainly supported by community and interdisciplinary strategies, with great potential for the health system and its role as guarantors of people’s health.^45^ ^,^ ^48^ However, there is still a long way to go in the development of evaluations of these programs in order to address their weaknesses and take advantage of their strengths.

*Cooperation as a possibility. Interweaving experiences beyond (territorial and epistemological) borders* - In migration contexts, health strategies should recognise and consider the potential of the experiences and knowledge of migrants affected by Chagas, especially in relation to health care. Different initiatives have tried to incorporate the migrant presence in some way. An example of this is the *expert patient* program,^45^ that includes migrants affected by Chagas in health teams to be in charge of establishing links between health systems and communities and actively supporting detection and accompaniment during treatment. The mentioned initiatives are recent and require evaluations to assess, for example, the impact they have on the community in terms of agency, the ways in which different knowledge, realities and health models are recognised and articulated or the impact on the life quality of those affected. However, it is essential to develop joint strategies that allow, from a critical perspective, reviewing these processes of recognition with the aim of articulating them with health systems, since the inclusion of the migrant population in the programs by itself does not ensure the incorporation of other visions, knowledge or the development of intercultural strategies. This recognition is also an opportunity to question the paradigms and power relations from which health-illness is approached in health systems in countries receiving migrants.

On the other hand, the social organisation and active participation of groups of people affected by Chagas at national and international level enhance dialogue and the possibilities of social and political incidence in favor of better conditions of access to health and guarantee of rights at global level.^44^ ^,^ ^46^ ^,^ ^48^ For technical teams, social organisation becomes an interlocutor to promote social and even political changes at the sectoral level. Likewise, cooperation between states and inter-institutional coordination, taking into account the health needs of cross-border communities, will benefit both the people who come and go between these territories and the health systems that care for them. Considering strategies that enhance participation, cooperation between states and health systems in territories where migratory circularity occurs is an opportunity to safeguard the health of people living and working in these areas,^31^ with special attention to those in a more vulnerable situation such as women of childbearing age, pregnant women and their children, and migrant workers. This cooperation would allow increased diagnosis, treatment and follow-up, especially in territories that respond to Chagas with different health policies and coverage.

Both the review of the interaction within health systems and the development of strategies and content to address Chagas should be built on intercultural bases aimed at improving the health and living conditions of migrants.

**Addressing the challenges of the coming decades in light of the opportunities ahead**

Migrants and refugees face complex problems that must be addressed cross-culturally, with an intersectional and collaborative approach among countries. The prevention and treatment of problems affecting people’s health is the responsibility of states and not only of those who suffer from them. Thus, access to health, regardless of nationality, origin or ethnicity, or individuals’ regularity status must be guaranteed by countries.

According to Glick-Schiller,^49^ “a multi-scalar global perspective asks scholars and policy makers to recognise that each state in the world represents the confluence in place and time of the multiple series of intersecting networks that can be analysed as transnational social fields. Migration scholars must understand that both immobility and mobility are interrelated dynamics that are activated and differently perceived and valued within the cross-border regimes that constitute these fields”. In other words, the global multi-scalar approach allows overcoming dichotomous constructions, revising, for example, the traditional endemic/non-endemic distinction, recognising the barriers and structural conditions of exclusion and inclusion, and urban, rural and regional differences; it also implies recognising migrants’ contributions, identifying their agency, their capacities to create new social practices and to have a political impact.

Undoubtedly, it is essential to develop intersectoral policies that promote collaboration and integration in health at national and regional levels and between border areas or cross-border communities; that is, large-scale integration programs are required to address migrants and refugees’ needs. The right to health must be addressed without discrimination of any kind, for which it is essential that countries adapt their legal frameworks and their police and border control systems, modify health systems towards more inclusive ones, strengthen public policies and increase migrants’ social and citizen participation. Universal access to health must guarantee people both equity in access to health services and quality of care and protection, regardless of the cost of such services.

Health intervention and IEC strategies, from an intercultural and intersectional perspective, enrich the possible responses and recognise the complexity and multidimensionality of Chagas in the migrant population. IEC strategies also reach the general population to avoid discrimination associated with Chagas disease, especially for people who - due to the conditions of migration (internal, international) - are particularly exposed to the violation of their rights.

A multidimensional approach^3^ allows addressing both Chagas with migrants and health teams linked to them, facilitating work in diverse sociocultural environments, in heterogeneous territories and institutions, and with different social actors and political scenarios. The sociocultural diversities associated with human mobility should be embodied in intercultural approaches that critically reflect on hegemonic medical practices; that is, those that favor the questioning of historical colonial frameworks and ethnocentric practices that prevent addressing diversity in global health contexts. This is an opportunity to interact and co-create from diverse knowledge and stimulate creative and integral approaches that favor the transformation of health realities. “In each of us there is a common Chagas, but at the same time it is a different Chagas for each of us. New stories are born wherever we go, and new Chagas, with possible hopes.” In light of these words, shared by a migrant woman who has Chagas disease and lives in Catalonia, we believe that we can face the coming years with optimism based on the following recommendations:

(i) The right to mobility, migration and refuge of people must be guaranteed. Each nation must ensure the appropriate ways of migratory regularisation and access to health, considering the protection and guarantee of labor and health’s rights of migrants at the international level.

(ii) In cross-border communities, intersectoral and international cooperation strategies should be developed to guarantee access to health care for the population. In particular, in relation to Chagas, progress should be made in comprehensive programs that ensure environmental conditions, vector control and access to health under equal conditions in territories that share borders to reduce the gaps and inequalities between them and between populations.

(iii) Health policy in receiving countries should consider human mobility as a key factor in the social determination of health and thus make progress in the detection, care and health follow-up of people affected by Chagas, highlighting the importance of including it as a universal screening infection in health services and building the capacity of these services so that care for Chagas can be provided at PHC level. Particular attention should be paid to women of reproductive age and pregnant women and their children to reduce inequalities and guarantee timely access to diagnosis and treatment.

(iv) Countries should collaborate and guarantee cooperation strategies that promote a comprehensive approach to advance towards health as a universal right.

(v) IEC is an opportunity to promote the autonomy of communities. In this endeavor, the transnational space is also an opportunity for the development of IEC strategies.

(vi) Chagas, in its intersection with people’ mobility, is a juncture for the review and transformation of mechanisms that ensure people’s access to health and that consider the particularities of aspects such as social class, life cycle, gender and educational level, among others.

(vii) To understand the diversity that emerges from the intersection between Chagas and human mobility, it is necessary to incorporate both the clinical and epidemiological analysis of this intersection, as well as the socio-cultural representations of the health-disease-care processes and of mobility and Chagas disease itself. It is also essential to consider the subjective, experiential and emotional dimensions related to Chagas and migration to address the design of truly comprehensive health policies and strategies.

In summary, it is essential to question the power positions of the different social actors and to recognise the socio-cultural processes on the basis of which the different health models and systems, including the biomedical one, are constructed. Approaches are required that dare to explore and orient themselves towards positions of symmetry in contexts of diversity through creative, sensitive, respectful and intercultural resolution strategies.

Today, Chagas is a global reality, which poses a great number of challenges that add to and make more complex the approaches needed to face the problem. In this framework, a multidimensional approach to Chagas allows us to question the constructions of health/disease, to approach diverse experiences based on human mobility and to critically consider the dichotomies of legal/illegal, poor/non-poor, developed/non-developed, endemic/non-endemic, rural/urban, etc., dichotomies through which the diversity of realities is blurred, as well as the particularities of Chagas and its possibilities of overcoming in each context.
